# Spermidine/spermine N1-acetyltransferase1mediates zearalenone-induced toxicity in bovine Leydig cells

**DOI:** 10.3389/fcell.2026.1934107

**Published:** 2026-09-11

**Authors:** Hongxia Li, Liwei Huang, Feifei Song, Xiaolong Pan, Guoqing Zhao, Juan Xiong, Pengkang Song, Le Zhao, Xuanqi Yu, Junyao Zhang, Ruojun Zong, Xi Wang, Ruigao Song

**Affiliations:** 1 Shanxi Bethune Hospital, Shanxi Academy of Medical Sciences, Third Hospital of Shanxi Medical University, Tongji Shanxi Hospital, Taiyuan, China; 2 Third Hospital of Shanxi Medical University, Shanxi Bethune Hospital, Shanxi Academy of Medical Sciences, Tongji Shanxi Hospital, Taiyuan, China; 3 College of Animal Science, Shanxi Agricultural University, Taigu, China; 4 College of Life Sciences, Shanxi Agricultural University, Taigu, China; 5 State Key Laboratory of Organ Regeneration and Reconstruction, Institute of Zoology, Chinese Academy of Sciences, Beijing, China

**Keywords:** apoptosis, bovine Leydig cells, oxidative stress, testosterone synthesis, zearalenone

## Abstract

**Introduction:**

Zearalenone (Zea), a widespread mycotoxin, can impair male reproductive function, but its effects on bovine Leydig cells and the underlying mechanisms remain insufficiently understood.

**Methods:**

Primary bovine Leydig cells were isolated and identified. The effects of Zea on cell viability, testosterone production, steroidogenic gene expression, oxidative stress, apoptosis, and cell cycle progression were examined. Transcriptomic analysis was performed to identify genes associated with Zea-induced cellular injury, and the role of spermidine/spermine N1-acetyltransferase1 (SAT1) was investigated using siRNA-mediated knockdown.

**Results:**

Zea exposure decreased cell viability in a concentration-dependent manner, with an approximate half-maximal inhibitory concentration of 75 μM, and reduced testosterone production and steroidogenic gene expression. Zea markedly increased intracellular reactive oxygen species levels and lipid peroxidation while suppressing antioxidant enzyme activities. It also promoted apoptosis and induced G2/M phase arrest. SAT1 was identified as one of the most upregulated genes following Zea exposure. SAT1 knockdown attenuated oxidative stress and apoptosis, restored antioxidant capacity, and improved cell proliferation under Zea exposure.

**Discussion:**

These findings demonstrate that SAT1 is involved in Zea-induced oxidative stress and cellular injury in bovine Leydig cells and provide new insight into the molecular basis of mycotoxin-induced reproductive toxicity.

## 1 Introduction

Zearalenone (Zea), a non-steroidal mycotoxin produced by Fusarium species, is widely detected in contaminated feed and poses a persistent threat to livestock health (Hai et al., 2023; Ning et al., 2026; Liu T. et al., 2023). Owing to its structural similarity to 17β-estradiol, Zea exerts pronounced estrogenic activity, particularly targeting the male reproductive system (Ning et al., 2026; Liu T. et al., 2023; Cao et al., 2021; Yang et al., 2026; Guo et al., 2025). Increasing evidence indicates that exposure to Zea disrupts steroidogenesis, impairs spermatogenesis, and compromises fertility in mammals, with Leydig cells being especially vulnerable (Hai et al., 2023; Ning et al., 2026; Chen et al., 2020).

Leydig cells are the primary source of testosterone synthesis in the testis and play a central role in maintaining male reproductive function (Ning et al., 2026; Mattos et al., 2023). Their physiological activity is tightly regulated by intracellular redox balance (Ning et al., 2026; Sies et al., 2024). Accumulating studies have demonstrated that Zea induces excessive reactive oxygen species (ROS) production, leading to oxidative stress, mitochondrial dysfunction, and ultimately cell cycle arrest or apoptosis (Hai et al., 2023; Ning et al., 2026; Cao et al., 2021; Chen et al., 2020; Ma et al., 2023; Fu et al., 2022; Liu L. et al., 2023). However, the molecular determinants linking oxidative stress to impaired Leydig cell function remain to be further elucidated.

Spermidine/spermine N^1^-acetyltransferase1 (*SAT1*) is a key rate-limiting enzyme in polyamine catabolism (Pegg, 2016), controlling intracellular polyamine homeostasis (Minois et al., 2011). Beyond its metabolic role, *SAT1* has emerged as an important regulator of oxidative stress and cell fate (Moinard et al., 2005). Previous work demonstrated that activation of *SAT1* promotes oxidative damage through enhanced polyamine catabolism and lipid peroxidation (Ou et al., 2016). Notably, polyamine metabolism has been increasingly linked to testicular function (Zhao et al., 2021), yet its involvement in mycotoxin-induced reproductive toxicity remains largely unexplored.

Given the established role of oxidative stress in Zea toxicity and the emerging function of *SAT1* in redox regulation, we hypothesized that *SAT1* contributes to Zea-induced Leydig cell injury. In this study, we investigated the effects of Zea on cell proliferation, cell cycle progression, and oxidative stress in bovine Leydig cells, with a particular focus on the role of *SAT1*. Our findings aim to provide mechanistic insight into mycotoxin-induced reproductive toxicity and identify potential molecular targets for intervention.

## 2 Materials and methods

### 2.1 Primary isolation and identification of Leydig cells

Animal tissue samples used in this study were collected from Wen Shui County, Lvliang City, Shanxi Province, and Guan Heng Meat Industry Co., Ltd. Three adult healthy bulls were selected from the company. Testicular tissue samples were obtained under aseptic conditions and immediately placed in a thermos flask containing 1× double-antibody saline solution for preservation. The samples were then rapidly transported to the laboratory for subsequent cell isolation procedures.

After tissue collection, remove the white membrane from the testis surface. Place approximately 1 cm^3^ of tissue in PBS (Biosharp, BL302A; Beijing, China) containing dual antibodies and wash until the supernatant becomes clear. Subsequently, mince the tissue, add 0.1% type IV collagenase (Sangon Bioengineering, A004186–0100; Shanghai, China), and digest at 37 °C for approximately 30 min, vigorously shaking every 5 min to prevent cell aggregation. After digestion, add complete medium (composed of 10% FBS (Keygen bio TECH, KGL3006-50; Nanjing, China), 1× penicillin-streptomycin dual antibiotics (NCM Biotech, C100C5; Suzhou, China), and DMEM/F12 basal medium (Gibco, C11330500BT; New York, USA)) to terminate the reaction. Filter the cell suspension through a 100 μm filter (BIOFIL, CSS013100; Guangzhou, China) and collect cells by centrifugation at 1500 rpm for 5 min. Resuspend the cell pellet in fresh medium and wash twice (1500 rpm, 5 min per wash), then resuspend in complete medium for subsequent use.

Cells were seeded onto coverslips placed in 24-well plates and cultured at 37 °C in 5% CO_2_ until attachment. The medium was removed, and cells were washed twice with PBS, followed by fixation in 4% paraformaldehyde for 15 min at room temperature. After two additional PBS washes, cells were permeabilized with 0.25% Triton X-100 (Biosharp, BS084; Beijing, China) for 10 min and washed three times (5 min each). Non-specific binding was blocked with 1% BSA (Solarbio, sw3015; Beijing, China) in PBST for 30 min. Primary antibodies (3β-HSD, Immunoway, YN0349; USA), diluted in 1% BSA/PBST, were applied and incubated overnight at 4 °C in a humidified chamber. Cells were then washed three times with PBS and incubated with the appropriate secondary antibody for 1 h at room temperature in the dark. Following three PBS washes, nuclei were counterstained using an anti-fluorescence quencher containing DAPI (Sven, SI103-12; Beijing, China). Fluorescence images were acquired using a fluorescence microscope.

### 2.2 Screening of zearalenone concentration

Cells were plated in 96-well plates and incubated for 12 h (37 °C, 5%CO_2_). The experimental setup included a blank group (medium only), a control group (cells with medium), and Zea-treated groups (Shanghai Yuanye Biotechnology, T92692; Shanghai, China) (25, 50, 75, 100, and 125 μM), each in triplicate. Following 24 h of Zea exposure, 10 μL of CCK-8 reagent (MCE, HY-K0301; New Jersey, U. S.) was added to each well and incubated for an additional 1 h. Absorbance values were measured using an enzyme microplate reader. Cell viability was calculated after blank subtraction and normalized to the control group. Based on the cell viability assay, 75 μM Zea was selected as the optimal treatment concentration for subsequent experiments, as it induced a significant reduction in cell viability while avoiding excessive cytotoxicity.

### 2.3 Testosterone level detection

Leydig cells were plated in 6-well plates and treated at about 75% confluence with either control and zearalenone (75 μM; n = 3 per group). After 24 h, supernatants were collected, centrifuged (3,000 rpm, 20 min), and used for testosterone quantification by ELISA (Wuhan Genmei, JYM0282Bo; Wuhan, China) following the manufacturer’s protocol.

### 2.4 Total RNA extraction and reverse transcription

Cells were harvested for total RNA extraction. Briefly, cells were washed once with PBS, followed by the addition of 1 mL TRnaZol Reagent (Xin Sai Mei, M5101; Suzhou, China) per well and a short incubation at room temperature. Total RNA was subsequently isolated according to the manufacturer’s protocol.

cDNA synthesis was performed using the SPARKscript II All-in-one RT SuperMix for qPCR (SparkJade, AG0305; Shandong, China) following the manufacturer’s instructions. The reaction system and thermal cycling conditions are detailed in Supplementary Table 1-1 and Supplementary Table 1-2, respectively.

### 2.5 Quantitative real-time PCR

qRT-PCR analysis was conducted using 2×SYBR Green qPCR Mix (SparkJade, AH0104, Shandong, China). The reaction system was prepared as described in Supplementary Table 1-3, transferred into qPCR tubes, and briefly centrifuged before amplification. The thermal cycling conditions are summarized in Supplementary Table 1-4. The primers used in this study were synthesized by Shengong Bioengineering (Shanghai, China); their sequences are listed in Supplementary Table 1-5. Relative mRNA levels were determined using the 2^−ΔΔCT^ method.

### 2.6 ROS determination

Leydig cells were grown on coverslips in 24-well plates and divided into control and zearalenone (Zea, 75 μM) groups. After 24 h of treatment, intracellular ROS levels were examined. ROS was detected using a DCFH-DA probe (Beyotime, S0033S; Shanghai, China). The probe was diluted (1: 1000) in serum-free medium to a final concentration of 10 μM. Cells were rinsed once with serum-free medium and then incubated with 250 μL of the probe solution at 37 °C, 5% CO_2_ for 20 min. Excess probe was removed by washing the cells three times. After staining, nuclei were counterstained with DAPI using an anti-fade mounting medium. Coverslips were mounted and immediately observed under a fluorescence microscope. Images were analyzed with ImageJ, and mean fluorescence intensity was calculated to reflect ROS accumulation.

### 2.7 Detection of glutathione peroxidase (GSH-Px)

Cells were lysed and centrifuged, and the supernatants were collected for subsequent analysis. Glutathione peroxidase (GSH-Px) activity was determined using a commercial assay kit (Bioswamp, BTK148; Wuhan, China) according to the manufacturer’s instructions. The assay consisted of two sequential steps: an enzymatic reaction system (Supplementary Table 1-6) followed by a colorimetric detection system (Supplementary Table 1-7). Reactions were performed in 96-well plates, including blank, background, and sample wells.

Reagents were added according to Supplementary Table 1-7, mixed thoroughly, and incubated at room temperature for1min. Absorbance was measured at 422 nm using a microplate reader with ddH_2_O as the reference. Activity values were calculated according to the kit instructions.

### 2.8 Detection of total superoxide dismutase (SOD)

Cells were washed with cold PBS after medium removal and lysed in SOD (Bioswamp, BTK032; Wuhan, China) extraction buffer. Following centrifugation (12, 000×g, 5min, 4 °C), supernatants were collected for analysis. The WST-8/enzyme working solution (160 μL/reaction) was prepared at a 151:8:1 ratio of assay buffer, WST-8 reagent, and enzyme solution. The reaction initiation solution was obtained by diluting the 16× stock 1:15 with assay buffer. Reactions were assembled in 96-well plates as specified (Supplementary Table 1-8), mixed, and incubated at 37 °C for 30 min. Absorbance was measured at 450 nm.

### 2.9 Detection of malondialdehyde (MDA) levels

Cells were lysed in RIPA buffer and centrifuged at 12, 000×g for 10 min to obtain the supernatant. The malondialdehyde (MDA) assay kit (Bioswamp, BTK022; Wuhan, China) was used in accordance with the manufacturer’s instructions. TBA stock solution (0.37%) was prepared by heating at 70 °C to facilitate dissolution. The MDA working solution (203 μL per reaction) consisted of TBA diluent (150 μL), TBA stock solution (50 μL), and antioxidant (3 μL). Reaction mixtures were assembled in 1.5 mL tubes (blank, standard, and sample) following Supplementary Table 1-9, mixed, and incubated at 100 °C for 15 min. After cooling, samples were centrifuged at 1, 000×g for 10 min. An aliquot (200 μL) of the supernatant was transferred to a 96-well plate, and absorbance at 532 nm was recorded. MDA content was calculated and normalized to protein concentration.

### 2.10 TUNEL staining

Cells grown on coverslips in 24-well plates were treated at about 60% confluence with Zea (75 μM) and control (n = 3). After 24 h, cells were fixed in 4% paraformaldehyde (4 °C, 30 min) following PBS washes, and then permeabilized with 0.2% Triton X-100 for 30 min. TUNEL staining (Biopmk, PMK0997; Hubei, China) was performed according to standard procedures. Briefly, cells were equilibrated, incubated with the TdT reaction mixture at 37 °C for 1 h in the dark, and washed thoroughly. Samples were further rinsed in PBS containing 0.1% Triton X-100 and 5 mg/mL BSA. Nuclei were counterstained with DAPI and mounted with antifade medium. Fluorescence images were captured and analyzed using ImageJ.

### 2.11 Apoptosis detection by annexin V-EGFP/PI double staining

Cells were seeded in 6-well plates and cultured until reaching approximately 75% confluence. The cells were then assigned to a control group and a zearalenone (Zea, 75 μM) treatment group, with three replicates per group, and incubated for 24 h. Following treatment, culture supernatants were collected, and the cells were washed once with PBS. Cells were subsequently detached using EDTA-free trypsin and centrifuged at 300 *g* for 5 min. The cell pellets were washed twice with PBS and resuspended in 500 μL of binding buffer. Annexin V-EGFP (5 μL) and propidium iodide (5 μL) (KeyGEN Bio TECH, KGA1101; Nanjing, China) were added, followed by incubation for 10 min at room temperature in the dark. Apoptosis was then analyzed using flow cytometry.

### 2.12 Western blot

Cells were seeded in 6-well plates and cultured to approximately 75% confluence, followed by treatment (n = 3). Cells were lysed in RIPA buffer containing PMSF on ice, and lysates were clarified by centrifugation (12,000 rpm, 5 min, 4 °C). The supernatant was denatured with 5× loading buffer (Solarbio, P1043) and heated to 95 °C for 5 min. Equal amounts of protein were resolved by SDS–PAGE (Cinotoni, AC0565; Changsha, China) and transferred onto membranes. Membranes were blocked, incubated with primary antibodies overnight at 4 °C, and then with secondary antibodies for 1 h at room temperature. Apply the ECL luminescent solution (Coolaber, SL1352) to the membrane. The membrane was scanned using a chemiluminescence imaging system (Servicebio, SCE-W3000). Antibody details are provided in Supplementary Table 1-10.

### 2.13 EDU staining

Cells were plated on coverslips in 24-well plates and allowed to reach about 60% confluence before treatment with zearalenone (75 μM) for 24 h (n = 3). Cell proliferation was assessed using an EdU incorporation assay (Beyotime, C0078S; Shanghai, China). Briefly, cells were incubated with 10 μM EdU for 2 h at 37 °C, followed by fixation in 4% paraformaldehyde and permeabilization with 0.3% Triton X-100. The Click reaction was performed according to the manufacturer’s instructions, and nuclei were counterstained with DAPI. After mounting, images were captured using a fluorescence microscope and analyzed with ImageJ.

### 2.14 Flow cytometry cell cycle detection

Cells were plated in 6-well plates and cultured to approximately 75% confluence prior to treatment. Cells were then exposed to zearalenone (75 μM) and maintained as untreated controls for 24 h, with three independent replicates per condition. After treatment, cells were collected by EDTA-free trypsin digestion (Servicebio, G4002; Wuhan, China), washed with PBS, and fixed in 70% cold ethanol at 4 °C overnight. Fixed cells were subsequently washed and stained with PI/RNase A working solution (1: 9) (KeyGEN Bio TECH, KGA9101; Nanjing, China) for 30 min at room temperature in the dark, followed by flow cytometric analysis.

### 2.15 RNA sequencing (RNA-seq)

Cells were plated in 6-well plates and cultured until approximately 75% confluence. Cells were then treated with 75 μM zearalenone (Zea) and control, with three replicates per condition. After 24 h, cells were washed once with PBS and lysed directly in TRIzol for sample collection. All samples were shipped on dry ice to Shanghai Sangon Biotech Co., Ltd. For RNA sequencing.

RNA sequencing was performed by Sangon Biotech (Shanghai, China). Briefly, total RNA was extracted, quality assessed, and used for mRNA library preparation. Libraries were constructed using an mRNA-seq workflow involving poly(A) enrichment, RNA fragmentation, cDNA synthesis, adaptor ligation, and PCR amplification. The qualified libraries were sequenced on the DNBSEQ-T7 platform using paired-end 150 bp sequencing.

Raw sequencing reads were filtered using FastQC and Trimmomatic (v0.36) to remove adaptor contamination, low-quality bases (Q < 20), and reads shorter than 35 bp. Clean reads were mapped to the bovine reference genome using HISAT2 (v2.0). Transcript expression levels were calculated using StringTie (v1.3.3b) and normalized as TPM values.

Differential expression analysis was conducted using DESeq2 (v1.12.4). Genes with q-value ≤0.001 and |fold change| ≥2 were defined as differentially expressed genes. GO and KEGG enrichment analyses were performed to identify biological processes and pathways associated with DEGs, with FDR <0.05 considered statistically significant.

### 2.16 The silence of *SAT1*

siRNA-mediated knockdown of *SAT1* was performed using Lipofectamine 3000 (Thermo Fisher Scientific, L3000015; United States). Two candidate siRNAs (*si-SAT1-462* and *si-SAT1-79*) were designed and synthesized by Shanghai Shengong Biotechnology (Supplementary Table 1-11). Cells were seeded in 6-well plates and transfected upon reaching approximately 70% confluence. Three experimental groups were established (NC, *si-SAT1-462*, and *si-SAT1-79*), each with three biological replicates. For transfection, Lipofectamine 3000 (7.5 μL) was diluted in 125 μL Opti-MEM (Gibco, 31985-062; New York, USA), while siRNA (10 μL, 20 μM) was diluted separately in 125 μL Opti-MEM. The solutions were combined, mixed gently, and incubated for 15 min at room temperature to allow complex formation. Cells were washed with PBS and incubated with Opti-MEM before the addition of transfection complexes. After 8 h, the medium was replaced with complete culture medium. Cells were allowed to recover before RNA extraction to determine knockdown efficiency. Based on these results, the siRNA with optimal efficiency was used for subsequent mechanistic studies involving NC, NC + Zea, *si-SAT1*, and *si-SAT1*+Zea groups.

### 2.17 Measurement of intracellular polyamine levels by ELISA

Intracellular spermine (Animalunion Biotechnology Co., Ltd., Cat. No. LV80561; Shanghai, China) and spermidine (Animalunion Biotechnology Co., Ltd., Cat. No. LV80562; Shanghai, China) levels were determined using commercial bovine polyamine ELISA kits according to the manufacturer’s instructions. Briefly, bovine Leydig cells from different experimental groups were collected after zearalenone treatment and *SAT1* knockdown. Cells were lysed, and the supernatants were obtained by centrifugation for subsequent analysis. The prepared samples and corresponding standards were added to ELISA plates and incubated under the conditions recommended by the manufacturer. After incubation with detection antibodies and enzyme conjugates, the chromogenic substrate was added for color development. The reaction was terminated, and absorbance was measured at 450 nm using a microplate reader. The concentrations of spermidine and spermine were calculated according to the standard curves generated from the corresponding standards. All measurements were performed with biological replicates.

### 2.18 Statistical analysis of the data

Data are expressed as mean ± SD from three independent experiments. Statistical analyses were performed using GraphPad Prism 9.0 (GraphPad Software, San Diego, CA, USA). Data distribution normality was assessed using the Shapiro–Wilk test, and homogeneity of variances was evaluated using the Brown–Forsythe test before statistical comparisons. For comparisons between two groups, an independent samples t-test was performed. For comparisons among multiple groups, one-way analysis of variance (ANOVA) followed by Tukey’s multiple comparisons test was used. Statistical significance was defined as *P* < 0.05, and differences were considered highly significant when *P* < 0.01.

For transcriptomic analysis, DEGs were screened using DESeq2 with the criteria of FDR< 0.05 and |log_2_ (fold change)| ≥ 1. GO and KEGG enrichment analyses were performed to determine significantly enriched pathways.

## 3 Results

### 3.1 The Effects of Zea on the viability of bovine Leydig cells and testosterone synthesis

**FIGURE 1:**
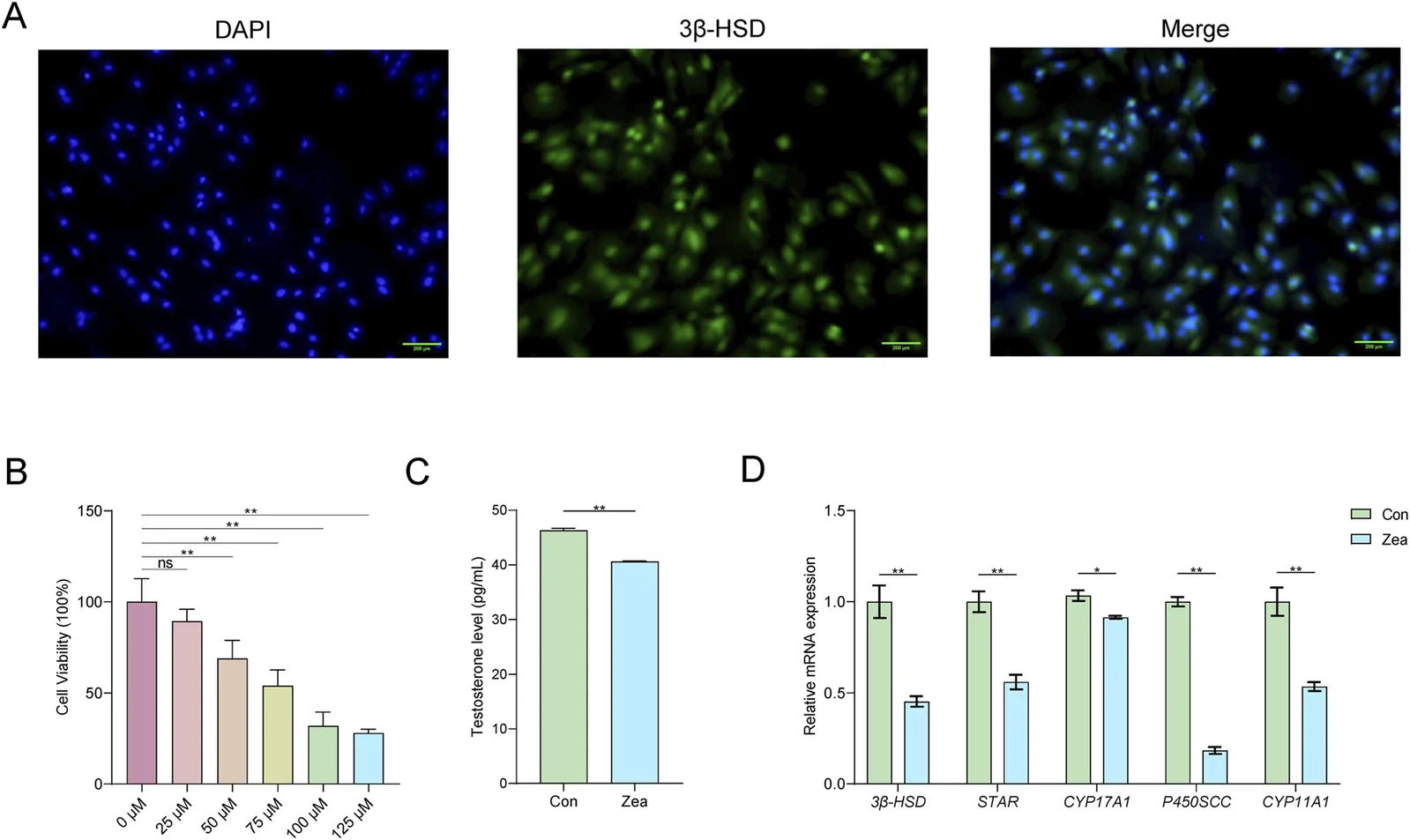
The Effects of Zea on the Viability of bovine Leydig Cells and Testosterone Synthesis. **(A)** Identification of bovine Leydig cells stained with 3β-HSD (scale bar 200 μM). **(B)** Changes in testis Leydig cell viability after treatment with different doses of Zea (*: *P* < 0.05; **: *P* < 0.01). **(C)** Testosterone levels. **(D)** mRNA expression levels of genes involved in testosterone synthesis.

Primary bovine Leydig cells isolated in our laboratory were identified by 3β-HSD immunofluorescence staining, with a purity of 92% ± 0.5% (n = 3) (Figure 1A; Table 1). CCK-8 analysis (Figure 1B) showed a significant, concentration-dependent decline in cell viability following zearalenone (Zea) exposure (*P* < 0.01), with a calculated CC_50_ of approximately 75 μM. Therefore, 75 μM Zea was selected as the working concentration for subsequent mechanistic investigations. This concentration was therefore selected for subsequent experiments. ELISA results (Figure 1C) demonstrated a marked reduction in testosterone levels in Zea-treated cells compared with controls (*P* < 0.01). Consistently, qPCR analysis (Figure 1D) revealed significant downregulation of genes involved in testosterone biosynthesis (*P* < 0.05). Together, these data indicate that Zea impairs both the viability and steroidogenic function of bovine Leydig cells.

**TABLE 1:** Purity percentage of bovine Leydig cells.

Category	DAPI-positive cells	3β-HSD-positive cells	Purity (%)
Figure 1	172	157	91%
Figure 2	207	194	94%
Figure 3	183	168	92%

### 3.2 The effect of Zea on oxidative stress in bovine Leydig cells

**FIGURE 2:**
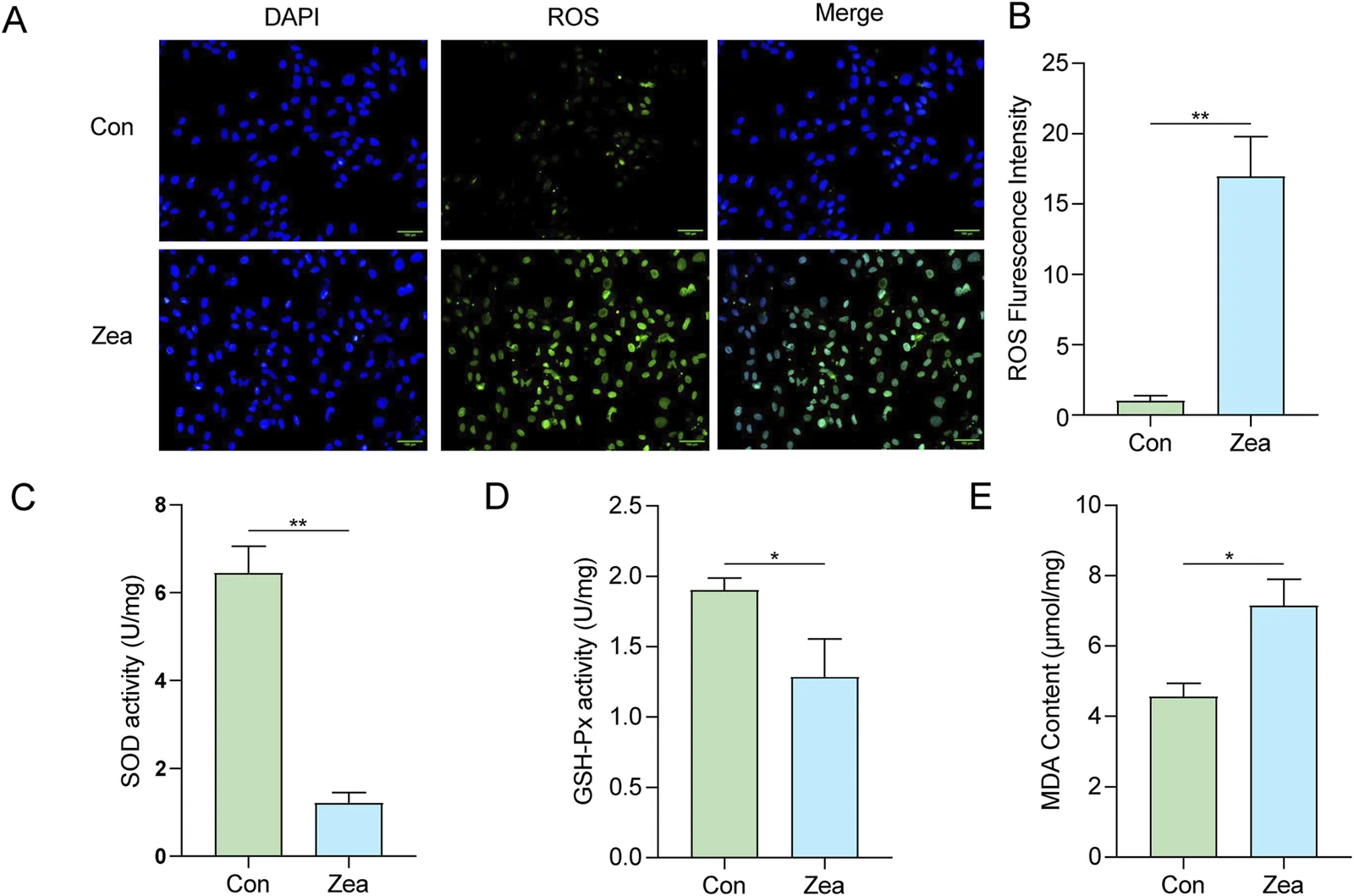
The effect of Zea on oxidative stress in bovine Leydig cells. **(A)** Immunofluorescence staining for ROS in Leydig cells (scale bar 100 μM). **(B)** Bar graph showing histogram of ROS fluorescence intensity (**: *P* < 0.01). **(C)** Zea effects on intracellular SOD activity (**: *P* < 0.01). **(D)** Zea effects on intracellular GSH-Px activity (*: *P* < 0.05). **(E)** Zea effects on intracellular MDA levels (*: *P* < 0.05).

As evidenced by ROS staining (Figures 2A,B), Zea treatment led to a pronounced accumulation of intracellular ROS (*P* < 0.01). In parallel, antioxidant enzyme activities SOD and GSH-Px were suppressed, whereas lipid peroxidation, reflected by MDA levels, was enhanced (Figures 2C–E; *P* < 0.05). Collectively, these results point to Zea-induced oxidative stress in bovine Leydig cells.

### 3.3 The effect of Zea on the apoptosis of bovine Leydig cells

**FIGURE 3:**
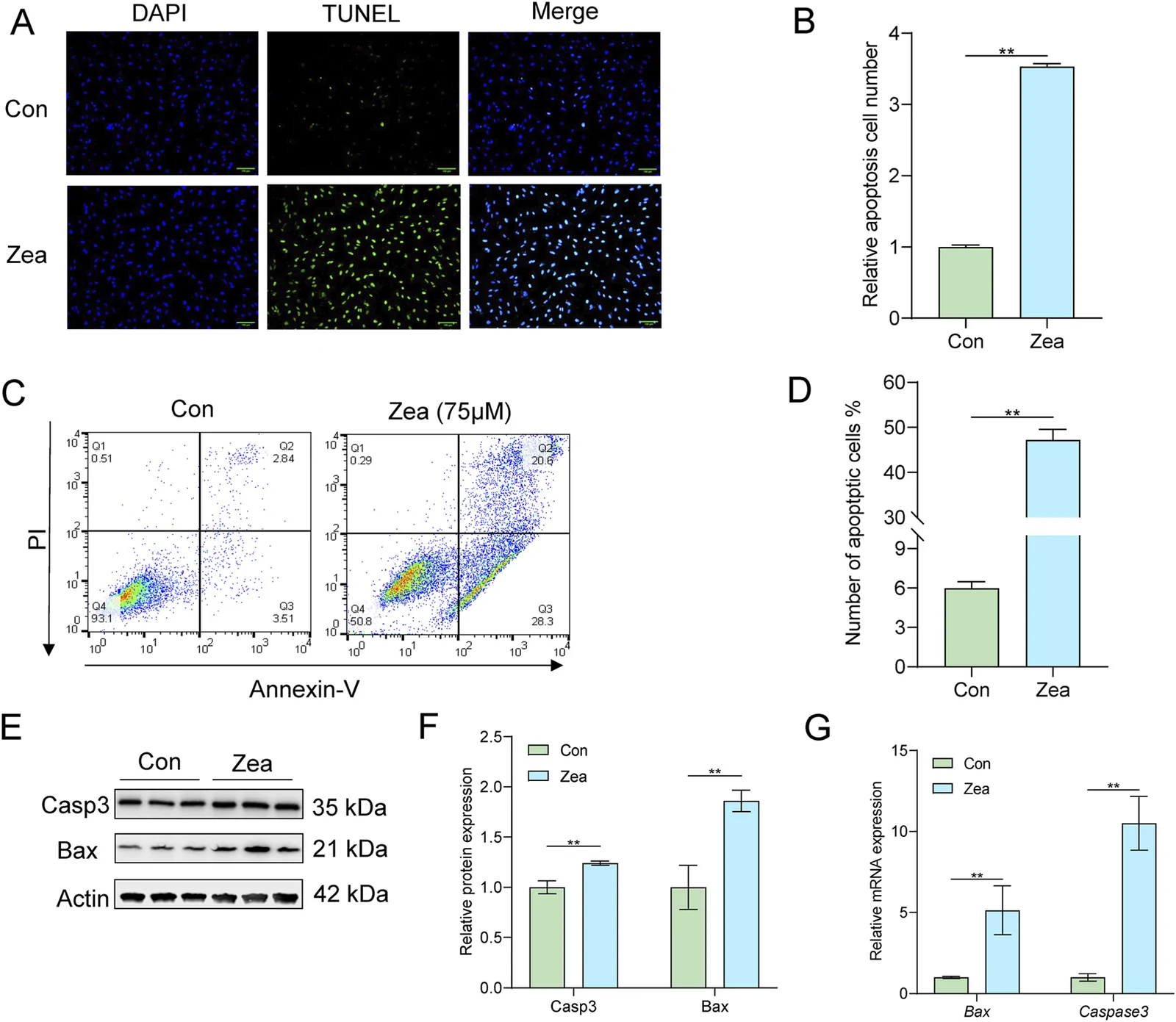
The effect of Zea on the apoptosis of bovine Leydig cells. **(A)** Leydig cells TUNEL staining (Scale bar = 100 μM). **(B)** Bar chart showing the number of apoptotic cells stained with TUNEL (**: *P* < 0.01). **(C)** Flow detection of apoptosis. **(D)** Flow Cytometry Graph Showing Apoptosis Rates (**: *P* < 0.01). **(E)** Expression levels of apoptosis-related proteins. **(F)** Analysis of Protein Gray Scale Values (**: *P* < 0.01). **(G)** mRNA expression levels of apoptosis-related genes (**: *P* < 0.01).

Apoptosis was assessed by TUNEL staining and flow cytometry (Figures 3A–D). Compared with the control, Zea treatment markedly increased apoptosis in bovine Leydig cells (*P* < 0.01). Western blot analysis (Figures 3E,F) showed that Zea significantly upregulated the protein levels of the pro-apoptotic markers Casp3 and Bax (*P* < 0.01). Consistently, qPCR results (Figure 3G) demonstrated a parallel increase in *Bax* and *Casp3* mRNA expression following Zea exposure (*P* < 0.01).

### 3.4 The Effect of Zea on the proliferation of bovine Leydig cells

**FIGURE 4:**
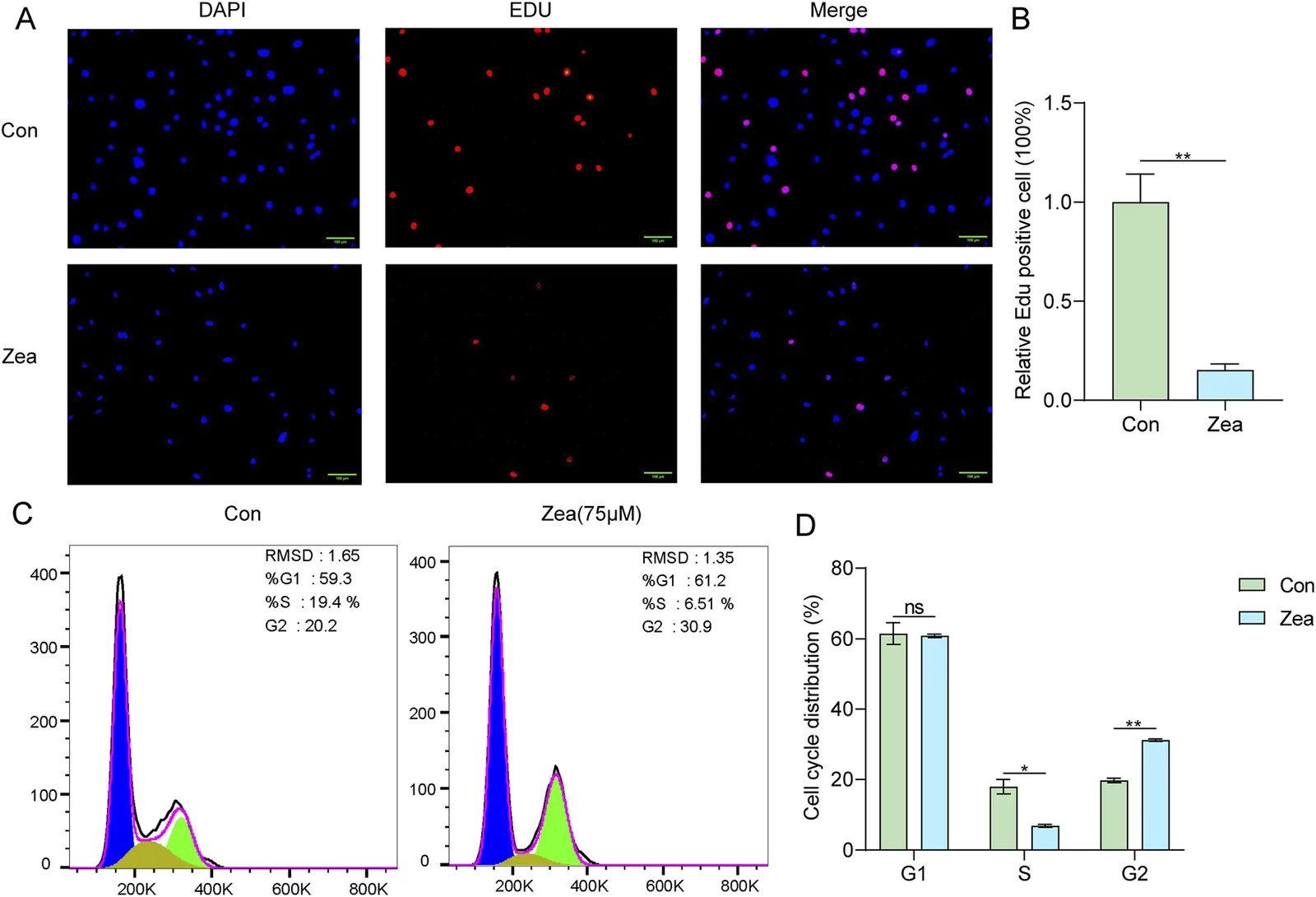
The Effect of Zea on the Proliferation of bovine Leydig cells. **(A)** Leydig cells stained with EDU (Scale bar = 100 μM). **(B)** EDU-stained cell positivity rate (**: *P* < 0.01). **(C)** Flow Cytometry for Cell Cycle Analysis. **(D)** Statistics on Cell Cycle Phases by Flow Cytometry (*: *P* < 0.05; **: *P* < 0.01).

EdU labeling (Figures 4A,B) demonstrated a significant reduction in DNA synthesis following Zea exposure (*P* < 0.01). Consistently, cell cycle profiling by flow cytometry (Figures 4C,D) showed a decrease in S-phase cells (*P* < 0.05) and a concomitant increase in the G2/M population (*P* < 0.01), suggesting that Zea induces arrest at the G2/M checkpoint.

### 3.5 Transcriptomic profiling reveals global gene expression alterations in Zea-treated bovine Leydig cells

**FIGURE 5:**
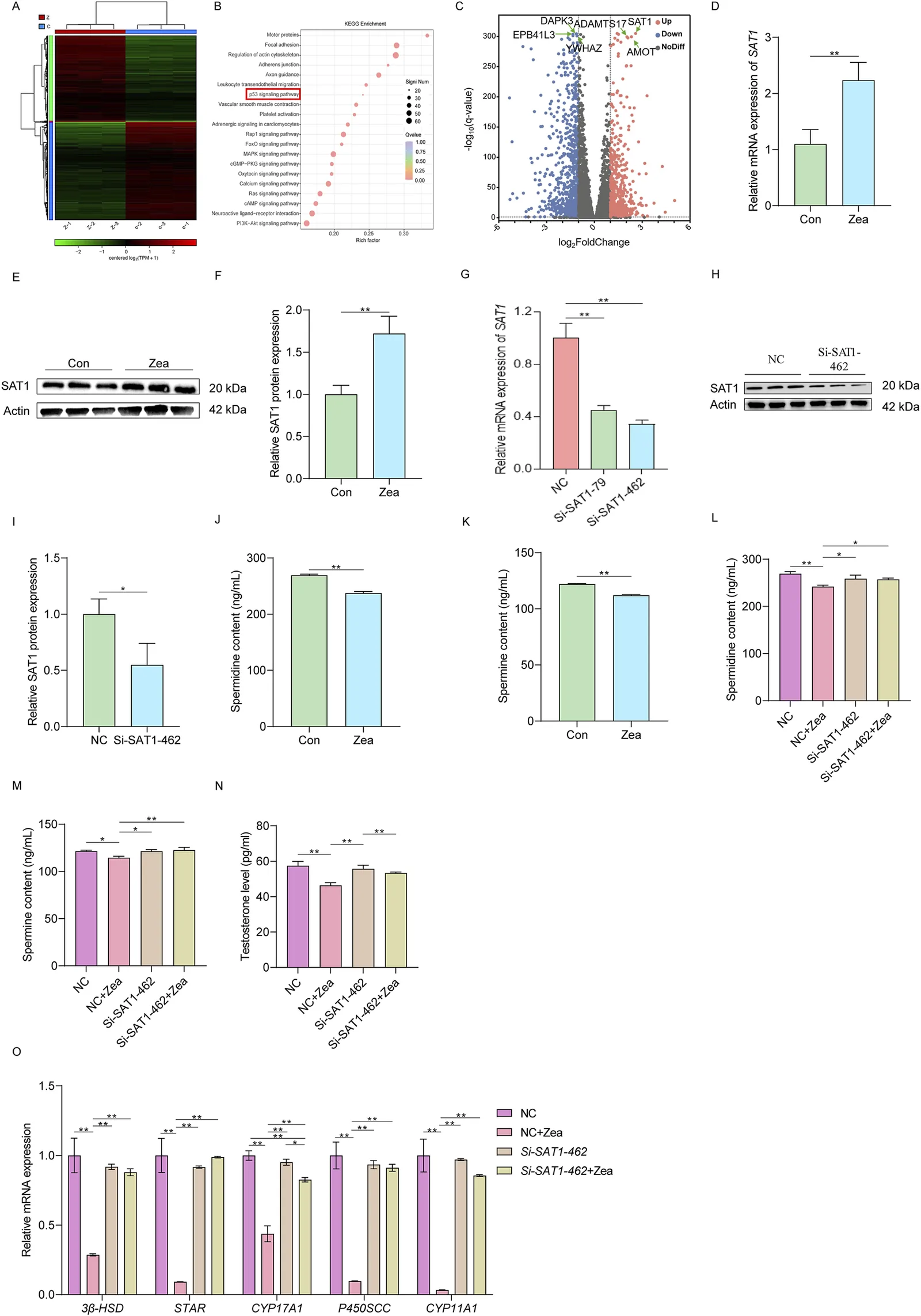
Transcriptomic profiling reveals global gene expression alterations in Zea-treated bovine Leydig cells. **(A)** Heat map of differential gene expression correlations between samples. **(B)** KEGG functional analysis of Zea-treated DEGs. **(C)** Volcano plots of differential gene expression between samples. **(D)** mRNA expression levels of the *SAT1* gene. **(E)** Protein expression levels of the SAT1 gene. **(F)** Protein grayscale analysis (**: *P* < 0.01). **(G)** Relative mRNA expression levels of *SAT1* (**: *P* < 0.01). **(H)** Protein expression levels of the SAT1 gene. **(I)** Protein grayscale analysis (*: *P* < 0.05). **(J)** Effect of Zea on intracellular spermidine levels (**: *P* < 0.01). **(K)** Effect of Zea on intracellular spermine levels (**: *P* < 0.01). **(L)** Effect of SAT1 on intracellular spermidine levels (*: *P* < 0.05; **: *P* < 0.01). **(M)** Effect of *SAT1* on intracellular spermine (*: *P* < 0.05; **: *P* < 0.01). **(N)** Testosterone levels (**: *P* < 0.01). **(O)** mRNA expression levels of genes involved in testosterone synthesis (*: *P* < 0.05; **: *P* < 0.01).

RNA-seq analysis revealed marked transcriptional alterations between Zea-treated and control Leydig cells (Figure 5A). A total of 2,832 differentially expressed genes were identified, including 1,324 upregulated and 1,508 downregulated genes. Functional enrichment analysis demonstrated significant involvement of these genes in the p53 signaling pathway (Figure 5B). Among the top upregulated genes identified by volcano plot analysis, *SAT1* showed a marked increase following Zea exposure (Figure 5C). qPCR and WB results further confirmed the elevated expression of SAT1 (Figures 5D–F, *P* < 0.01). Given that *SAT1* functions as a key regulator of spermidine/spermine catabolism, we next investigated whether *SAT1* affected intracellular polyamine homeostasis.

Two siRNAs targeting *SAT1* were designed, and *si-SAT1-462* exhibited the strongest knockdown efficiency and was selected for subsequent experiments (Figures 5G–I,P < 0.05; *P* < 0.01). Zea exposure significantly decreased intracellular spermidine and spermine levels (Figures 5J,K, *P* < 0.01), whereas *SAT1* silencing restored the abundance of these polyamines (Figures 5L,M, *P* < 0.05; *P* < 0.01). These results indicate that *SAT1* contributes to Zea-induced disruption of polyamine metabolism.

We further examined whether *SAT1* depletion affected Leydig cell steroidogenic function. Zea treatment significantly reduced testosterone production and downregulated the expression of steroidogenic genes, including *3β-HSD*, *STAR*, *CYP17A1*, *P450SCC*, and *CYP11A1* (Figures 1C,D, *P* < 0.05). In contrast, *SAT1* knockdown significantly rescued testosterone synthesis and restored steroidogenic gene expression despite continued Zea exposure (Figures 5N,O, *P* < 0.05; *P* < 0.01).

### 3.6 The effect of the *SAT1* gene on oxidative stress in bovine Leydig cells

**FIGURE 6:**
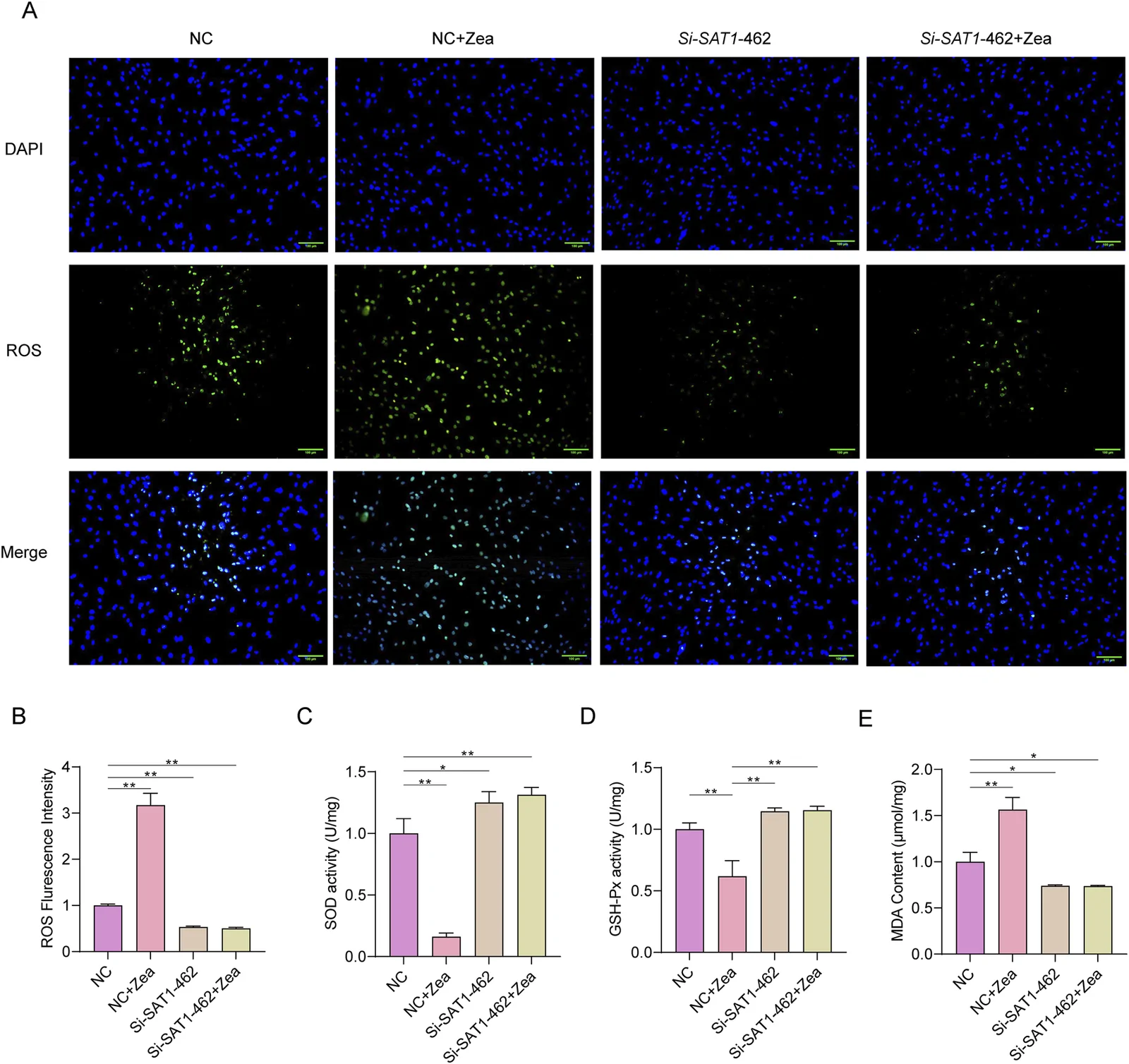
The Effect of the *SAT1* Gene on Oxidative Stress in bovine Leydig cells. **(A)** Immunofluorescence staining for ROS in Leydig cells (scale bar 100 μM). **(B)** Bar graph showing histogram of ROS fluorescence intensity (**: *P* < 0.01). **(C)** *SAT1* effects on intracellular SOD activity (*: *P* < 0.05; **: *P* < 0.01). **(D)** *SAT1* effects on intracellular GSH-Px activity **: *P* < 0.01). **(E)** *SAT1* effects on intracellular MDA levels (*: *P* < 0.05; **: *P* < 0.01).

As shown by ROS staining (Figures 6A,B), intracellular ROS levels were clearly reduced in the *si-SAT1-462* group compared with both NC and NC + Zea groups (*P* < 0.05). This reduction was still evident after Zea treatment, suggesting that loss of *SAT1* helps maintain redox stability even under stress conditions. Consistently, in the absence of Zea, SOD and GSH-Px activities were higher in the knockdown group, whereas MDA levels were lower than in controls (*P* < 0.05) (Figures 6C–E). After Zea exposure, these indices showed no further deterioration, indicating that oxidative damage was largely restrained when *SAT1* was suppressed. Taken together, the data suggest that *SAT1* acts upstream in the oxidative response to Zea, and its inhibition alleviates ROS accumulation while preserving antioxidant function.

### 3.7 The effect of the *SAT1* Gene on apoptosis in bovine Leydig cells

**FIGURE 7:**
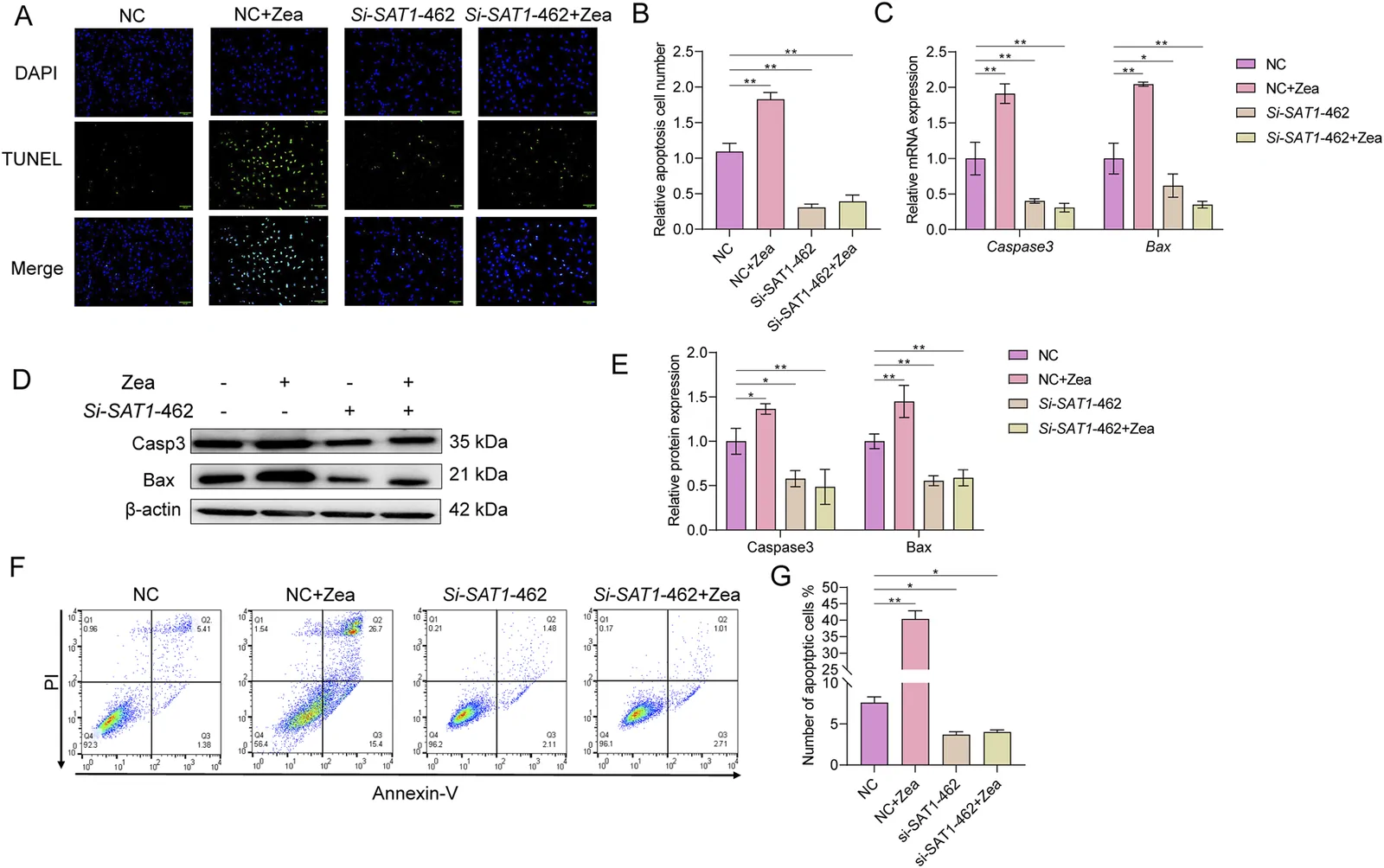
The Effect of the *SAT1* Gene on Apoptosis in bovine Leydig cells. **(A)** Leydig cells TUNEL staining (Scale bar = 100 μM). **(B)** Bar chart showing the number of apoptotic cells stained with TUNEL (**: *P* < 0.01). **(C)** mRNA expression levels of apoptosis-related genes (*: *P* < 0.05; **: *P* < 0.01). **(D)** Expression levels of apoptosis-related proteins. **(E)** Analysis of Protein Gray Scale Values (*: *P* < 0.05; **: *P* < 0.01). **(F)** Flow detection of apoptosis. **(G)** Flow Cytometry Graph Showing Apoptosis Rates (*: *P* < 0.05; **: *P* < 0.01).

TUNEL assays (Figures 7A,B) showed that, compared with their respective control groups (NC and NC + Zea), the number of apoptotic cells was significantly reduced in both the *si-SAT1*-462 group and the *si-SAT1*-462+Zea group (*P* < 0.05), and there was no significant difference between the *si-SAT1*-462 and *si-SAT1-462*+Zea groups. Similarly, Western blot and qPCR results indicated that, compared with the NC and NC + Zea groups, both mRNA (Figure 7C) and protein (Figures 7D,E) levels of Caspase-3 and Bax were significantly reduced in the *si-SAT1*-462 group (*P* < 0.05), but no changes were observed compared with the *si-SAT1*-462+Zea group. Flow cytometry (Figures 7F,G) further confirmed that the apoptosis rate was reduced following *SAT1* knockdown (*P* < 0.05). Taken together, these data indicate that *SAT1* knockdown attenuates Zea-induced apoptosis by suppressing the expression of pro-apoptotic genes and proteins.

### 3.8 The effect of the *SAT1* gene on the proliferation of bovine Leydig cells

**FIGURE 8:**
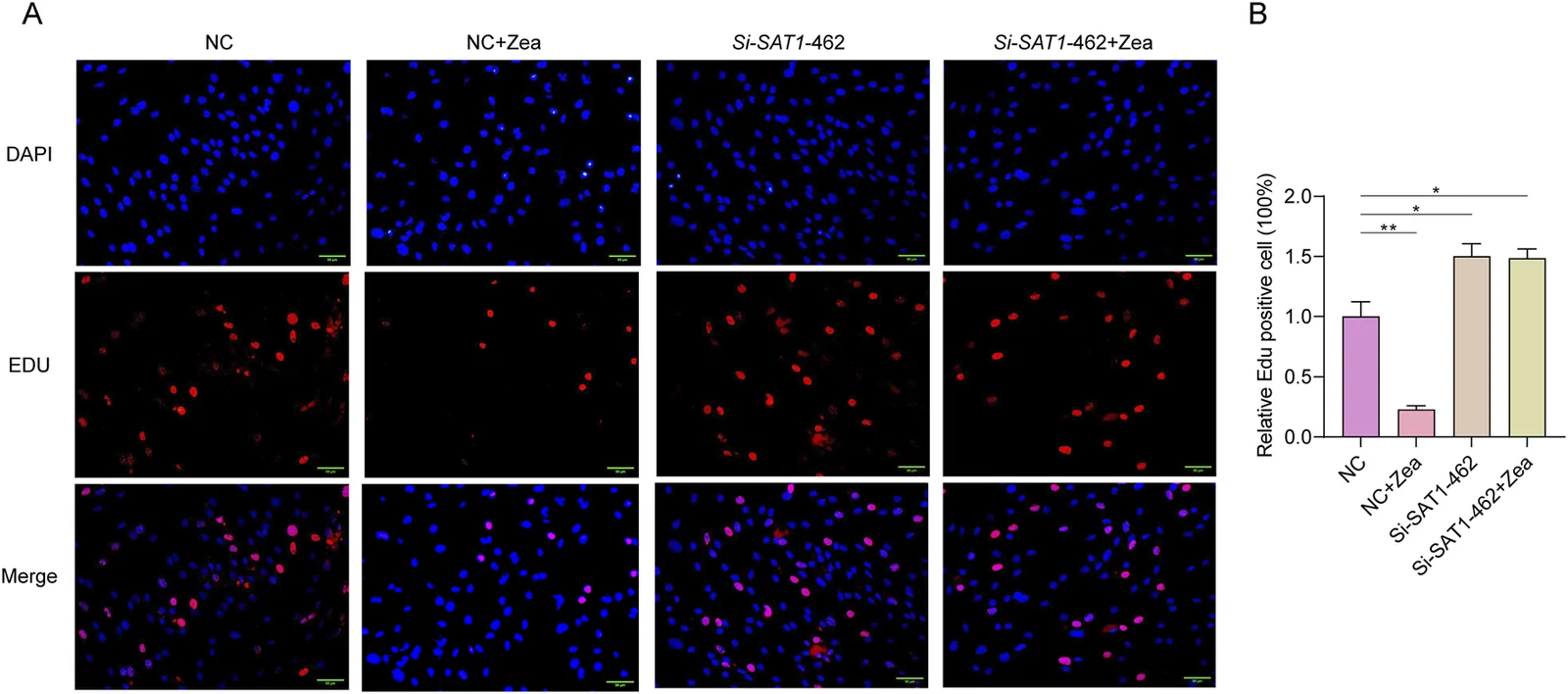
The Effect of the *SAT1* Gene on the Proliferation of bovine Leydig cells. **(A)** Leydig cells stained with EDU (Scale bar = 50 μM). **(B)** EDU-stained cell positivity rate (*: *P* < 0.05; **: *P* < 0.01).

As shown in Figures 8A,B, EdU staining revealed significantly higher proliferation in both the *si-SAT1*-462 and *si-SAT1*-462+Zea groups relative to the NC and NC + Zea groups (*P* < 0.05). In contrast, proliferation did not differ significantly between the two *si-SAT1*-462-treated groups, suggesting that silencing *SAT1* effectively counteracts Zea-induced suppression of cell proliferation.

## 4 Discussion

The present study demonstrates that zearalenone (Zea) exerts pronounced cytotoxic effects on bovine Leydig cells, characterized by reduced viability, impaired steroidogenesis, and disrupted cell cycle progression. These findings are consistent with recent reports showing that Zea compromises testicular function primarily through oxidative stress–dependent mechanisms (Ma et al., 2023; Liu X. et al., 2023; Zhao et al., 2026; Bai et al., 2022).

A central observation of this study is the marked accumulation of intracellular ROS following Zea exposure, accompanied by decreased antioxidant enzyme activities and enhanced lipid peroxidation. This redox imbalance is widely recognized as a key driver of Zea-induced reproductive toxicity (Bai et al., 2022; Yan et al., 2022). In Leydig cells, oxidative stress is particularly detrimental, as steroidogenesis is highly sensitive to mitochondrial integrity and redox status (Monageng et al., 2023; Wang et al., 2017). The observed suppression of testosterone synthesis and downregulation of steroidogenic genes in our study further support the notion that oxidative damage directly interferes with endocrine function.

Consistent with the redox disturbance, Zea exposure promoted apoptosis, as evidenced by increased TUNEL positivity and upregulation of Caspase-3 and Bax (Zhu et al., 2021; Hu et al., 2016; Lee et al., 2021). Oxidative stress–induced apoptosis has been extensively documented in testicular cells, where excessive ROS can trigger mitochondrial pathways leading to caspase activation (Chen et al., 2021; Finelli et al., 2022; Asadi et al., 2021; Nazanin et al., 2022). In parallel, we observed a reduction in DNA synthesis and a shift in cell cycle distribution, with a decrease in S-phase cells and accumulation in G2/M phase (Bai et al., 2023; Ji et al., 2022; Zheng et al., 2018; Wang et al., 2018). This pattern suggests activation of DNA damage checkpoints, a response commonly associated with oxidative stress and genomic instability (Bai et al., 2023; Yates et al., 2025).

Transcriptomic profiling demonstrated that Zea exposure induces substantial transcriptional reprogramming in Leydig cells, with numerous genes differentially expressed. Pathway enrichment analysis identified the p53 signaling pathway as a major node, suggesting engagement of canonical stress-response and damage-control mechanisms (Hernández Borrero and El-Deiry, 2021; Hafner et al., 2019). Given the pivotal role of p53 in coordinating oxidative stress and apoptotic signaling (Hao et al., 2023; Hanson and Batchelor, 2022), these data provide mechanistic support for Zea-induced cellular injury.

A notable finding of this study is the identification of *SAT1* as a potential upstream regulator of Zea-induced oxidative stress. Transcriptomic analysis revealed significant upregulation of *SAT1* following Zea exposure. As a key enzyme in polyamine catabolism, *SAT1* has been implicated in the generation of hydrogen peroxide and other reactive intermediates during polyamine turnover (Ou et al., 2016; Holbert et al., 2022; Murray Stewart et al., 2018). Recent studies have further linked *SAT1* activation to lipid peroxidation, highlighting its role in amplifying oxidative damage (Ou et al., 2016; Jiang et al., 2021). *SAT1* is a transcriptional target of p53, and its activation at the transcriptional level is typically accompanied by an increase in *SAT1* expression (Ou et al., 2016). In this context, p53-mediated induction of *SAT1* promotes polyamine catabolism, leading to enhanced generation of reactive oxygen species and thereby contributing to oxidative stress–associated cell death (Ou et al., 2016).

Functional validation through *SAT1* knockdown provided direct evidence for its involvement in Zea toxicity. Silencing *SAT1* effectively reduced ROS accumulation, restored antioxidant capacity, and limited lipid peroxidation, indicating that *SAT1* contributes to redox imbalance under Zea exposure (Ou et al., 2016). Moreover, inhibition of *SAT1* attenuated apoptosis and preserved proliferative capacity, suggesting that *SAT1* acts upstream of both oxidative stress and cell fate decisions (Ou et al., 2016; Mandal et al., 2015). These findings align with emerging evidence that polyamine metabolism is closely integrated with cellular stress responses and survival pathways (Ou et al., 2016; Thomas and Thomas, 2003; Schibalski et al., 2024).

Importantly, *SAT1* knockdown also mitigated Zea-induced cell cycle disruption, as reflected by improved DNA synthesis and normalized cell cycle distribution (Ou et al., 2016). These findings suggest that *SAT1*-mediated polyamine metabolism contributes to Zea-induced cell cycle dysregulation, potentially through mechanisms associated with oxidative stress responses. In addition to regulating cell proliferation, redox homeostasis is essential for maintaining Leydig cell steroidogenic function (Xiong et al., 2022). Excessive ROS accumulation can impair mitochondrial activity and consequently suppress testosterone biosynthesis by disrupting mitochondrial-dependent steroidogenic pathways (Jing et al., 2020). Consistent with this, our results demonstrate that SAT1deficiency significantly reduced Zea-induced accumulation of ROS and malondialdehyde (MDA) while enhancing antioxidant capacity—indicated by elevated levels of superoxide dismutase (SOD) and glutathione peroxidase (GSH-Px)—thereby effectively mitigating the Zea-induced decline in testosterone synthesis. Collectively, these findings suggest that *SAT1* promotes Zea-induced Leydig cell injury by disturbing redox balance and may subsequently impair mitochondrial-dependent steroidogenic processes. Given the critical role of Leydig cells in testosterone production, the protective effects of *SAT1* inhibition may have broader implications for maintaining male reproductive endocrine function under zearalenone exposure.

Taken together, our results support a model in which Zea induces oxidative stress in bovine Leydig cells, leading to apoptosis and cell cycle arrest, while *SAT1* functions as a key mediator that amplifies these effects through polyamine-dependent redox dysregulation. Targeting *SAT1* may therefore represent a potential strategy to mitigate zearalenone-induced reproductive toxicity.

This study explored the toxic mechanism of zearalenone (Zea) on bovine Leydig cells, constructed a Zea-induced oxidative stress and apoptosis model, and confirmed the core regulatory role of the *SAT1* gene in this process. This study is based on an *in vitro* cell model, which has significant advantages in researching molecular mechanisms, but its simulation of the complex physiological environment *in vivo* is still limited. This study found that the *SAT1* gene plays a key role in Zea-induced oxidative stress and apoptosis, but its specific upstream and downstream mechanisms remain unclear and still need further confirmation.

## 5 Conclusion

The present study showed that zearalenone impairs the viability and steroid synthesis function of bovine Leydig cells, mainly by inducing oxidative stress. Elevated levels of reactive oxygen species, impaired activity of antioxidant enzymes, and enhanced lipid peroxidation occur alongside increased apoptosis and cell cycle disruption, ultimately leading to decreased cellular function. Mechanistically, *SAT1* is a key regulator of this response. Even under zearalenone-exposed conditions, knockdown of *SAT1* attenuated oxidative damage, prevented excessive reactive oxygen species accumulation, and maintained antioxidant defenses. This stabilization of the redox state was accompanied by attenuation of apoptotic signaling and restoration of proliferative capacity. Zearalenone exposure induces *SAT1* overexpression in bovine Leydig cells, leading to excessive oxidative stress. Elevated *SAT1* expression subsequently suppresses steroidogenic capacity by downregulating key steroidogenic genes, including *3β-HSD*, *STAR*, *CYP17A1*, *P450SCC*, and *CYP11A1*, resulting in reduced testosterone synthesis. *SAT1* silencing alleviates oxidative damage, restores mitochondrial homeostasis, rescues steroidogenic gene expression, and partially restores testosterone production. These findings suggest that *SAT1*-dependent redox imbalance is a central mechanism of zearalenone-induced cellular damage. Therefore, targeting *SAT1* may be a viable strategy to protect bovine Leydig cell function from mycotoxin-induced oxidative damage.

## Data Availability

The original contributions presented in the study are publicly available. These data can be found in the NCBI BioProject database under accession number PRJNA1520498.
