# Supplementary material for: Spermidine/spermine N1-acetyltransferase1mediates zearalenone-induced toxicity in bovine Leydig cells
Source: Front Cell Dev Biol. 2026 Sep 11;14:1934107. doi: 10.3389/fcell.2026.1934107 (PMC13612519; doi:10.3389/fcell.2026.1934107)
Supplement: Supplementary file 1 [file Table1.docx]

Tables 1-1 System of reverse transcription reaction

| Component | Dose |
| --- | --- |
| Total RNA | 1 μg |
| gDNA Eraser | 1 μL |
| 2xSPARKscriptⅡAll-in-one RT SuperMix | 10 μL |
| RNase Free H_2_O | Up to 20 μL |

Tables 1-2 Reverse Transcription Protocol

| Temperature | Time |
| --- | --- |
| 50℃ | 15 min |
| 85℃ | 5 sec |

Tables 1-3 System of qRT-PCR reaction

| Component | Volume |
| --- | --- |
| 2xSYBR qPCR Mix | 5 |
| cDNA | 0.5 |
| Forward Primer（10 μM） | 0.2 |
| Reverse Primer（10 μM） | 0.2 |
| RNase Free H_2_O | 4.1 |

Tables 1-4 qPCR reaction protocol

| Temperature | Time | Number of iterations |
| --- | --- | --- |
| 94℃ | 3 min | 1 |
| 94℃ | 10 sec | 40 cycles |
| 60℃ | 34 sec |  |

Tables 1-5 Primer sequences of qRT-PCR

| Gene | Product length/bp | Primer sequence (5’-3’) |
| --- | --- | --- |
| *β-actin* | 148 | F：GCGGCATTCACGAAACTACC |
|  |  | R：GCCAGGGCAGTGATCTCTTT |
| *BCL-2* | 130 | F：ACGCCTTTGTGGAGCTGTAT |
|  |  | R：TTATGGCCCAGATAGGCACC |
| *Bax* | 159 | F：CAGCTCTGAGCAGATCATGAAGA |
|  |  | R：CCGATGCGCTTCAGACACT |
| *Caspase3* | 118 | F：TGAGGCAGACTTCTTGTACGC |
|  |  | R：GTGAGCGTGCTTTTTCAGCAT |
| *STAR* | 183 | F：CCCGAGACTTTGTGAGCGTA |
|  |  | R：GCCAGGTGAGTTTGGTCCTT |
| *P450scc* | 102 | F：GAAGAAAGACCGGGTGGTCC |
|  |  | R：AGGCTGACGAAGTCCTGAGA |
| *CYP17A1* | 154 | F：CTCCAGCATTGGCGACCTTA |
|  |  | R：TGATGGCGAGATGAGTTGCG |
| *CYP11A1* | 154 | F：CGCTCAGCCCTGGTCAAAG |
|  |  | R：ATTGCCAAGCTTCTCCCTGTAAAT |
| *3β-HSD* | 240 | F：AACTCGGGCTGCCTTTGACT |
|  |  | R：AGCAGCTGGGTACCTTTCACA |
| *SAT1* | 199 | F：GCTTAGTTGCAGAAGTGCCC |
|  |  | R：CACTTCATTGCAACCTGGCT |

Tables 1-6 GSH-Px enzymatic reaction system

| Add substance (mL) | Blank control tube | Background control tube | Measuring tube |
| --- | --- | --- | --- |
| GSH-Px Working Solution（1 mmol/L） | — | 0.02 | 0.02 |
| Test sample | — | — | 0.02 |
| ddH_2_O | 0.02 | 0.02 | — |
| Mix well and place in a 37°C water bath for 5 min. | | | |
| Oxidation bath (preheated to 37°C) | — | 0.01 | 0.01 |
| Mix well and place in a 37°C water bath for 5 min. | | | |
| Acid precipitation | 0.08 | 0.2 | 0.2 |
| Centrifuge at 3,500 g for 10 min. | | | |
| Collect the supernatant | — | 0.1 | 0.1 |

Tables 1-7 GSH-Px colorimetric reaction system

| Add substance (mL) | Blank control tube | Background control tube | Measuring tube |
| --- | --- | --- | --- |
| Collect the supernatant | — | 0.1 | 0.1 |
| Blank control | 0.1 | — | — |
| GSH-Px Assay Buffer | 0.125 | 0.125 | 0.125 |
| Benzoic acid color development solution | 0.025 | 0.025 | 0.025 |

Tables 1-8 Sample addition reagent

|  | Sample | Blank control well 1 | Blank control well 2 |
| --- | --- | --- | --- |
| Test sample | 20 µL | — | — |
| SOD Assay Buffer | — | 20 µL | 40 µL |
| WST-8/Enzyme Working Solution | 160 µL | 160 µL | 160 µL |
| Reaction initiator solution | 20 µL | 20 µL | — |

Tables 1-9 Sample addition reagent

|  | Blank control | Reference standard | Sample |
| --- | --- | --- | --- |
| RIPA lysis buffer | 0.1 mL | — | — |
| Reference standard | — | 0.1 mL | — |
| Test sample | — | — | 0.1 mL |
| MDA Assay Working Solution | 0.2 mL | 0.2 mL | 0.2 mL |

Tables 1-10 Antibody Information

| Antibody Name | Company |
| --- | --- |
| 3β-HSD | Immunoway |
| beta-Actin | Abways |
| Caspase-3 | Abways |
| Bax | Abways |
| SAT1 | Bioss |
| A horseradish peroxidase (HRP)-conjugated goat anti-rabbit secondary antibody | Abcom |

Tables 1-11 *SAT1* siRNA sequences

| siRNA | Sequence |
| --- | --- |
| si-*SAT1*-462 | Sense:5’- GACUCUUCAAGAUCGACAA/dT//dT/-3’ |
|  | Antisense:5’- UUGUCGAUCUUGAAGAGUC/dT//dT/-3’ |
| si-*SAT1*-79 | Sense:5’- CGAAUACAUGGAAGAGCAA/dT//dT/-3’ |
|  | Antisense:5’- UUGCUCUUCCAUGUAUUCG/dT//dT/-3’ |
| NC | Sense:5’- UUCUCCGAACGUGUCACGUTT-3’ |
|  | Antisense:5’- ACGUGACACGUUCGGAGAATT-3’ |
